# Determinants of Superselectivity—Practical Concepts for Application in Biology and Medicine

**DOI:** 10.1021/acs.accounts.2c00672

**Published:** 2023-03-14

**Authors:** Galina V. Dubacheva, Tine Curk, Ralf P. Richter

**Affiliations:** † Département de Chimie Moléculaire (DCM), UMR 5250, Université Grenoble Alpes, CNRS, 38000 Grenoble, France; ‡ Department of Materials Science and Engineering, Johns Hopkins University, Baltimore, Maryland 21218, United States; § School of Biomedical Sciences, Faculty of Biological Sciences, School of Physics and Astronomy, Faculty of Engineering and Physical Sciences, Astbury Centre for Structural Molecular Biology, and Bragg Centre for Materials Research, University of Leeds, Leeds, LS2 9JT, United Kingdom

## Abstract

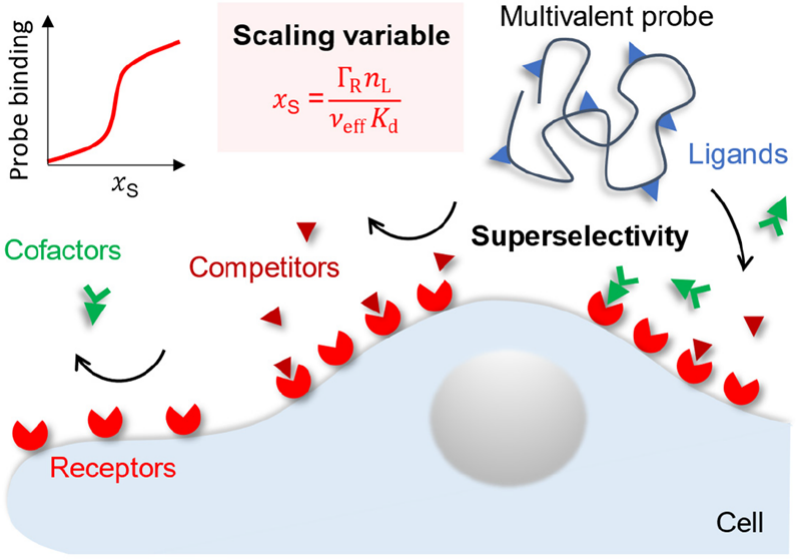

Multivalent interactions are common in biological systems and are also widely deployed for targeting applications in biomedicine. A unique feature of multivalent binding is “superselectivity”. Superselectivity refers to the sharp discrimination of surfaces (e.g., on cells or cell compartments) by their comparative surface densities of a given receptor. This feature is different from the conventional “type” selectivity, which discriminates surfaces by their distinct receptor types. In a broader definition, a probe is superselective if it converts a gradual change in any one interaction parameter into a sharp on/off dependency in probe binding.

This Account describes our systematic experimental and theoretical efforts over the past decade to analyze the determinants of superselective binding. It aims to offer chemical biologists, biophysicists, biologists, and biomedical scientists a set of guidelines for the interpretation of multivalent binding data, and design rules for tuning superselective targeting. We first provide a basic introduction that identifies multiple low-affinity interactions and combinatorial entropy as the minimal set of conditions required for superselective recognition. We then introduce the main experimental and theoretical tools and analyze how salient features of the multivalent probes (i.e., their concentration, size, ligand valency, and scaffold type), of the surface receptors (i.e., their affinity for ligands, surface density, and mobility), and of competitors and cofactors (i.e., their concentration and affinity for the ligands and/or receptors) influence the sharpness and the position of the threshold for superselective recognition.

Emerging from this work are a set of relatively simple yet quantitative data analysis guidelines and superselectivity design rules that apply to a broad range of probe types and interaction systems. The key finding is the scaling variable *x*_S_ which faithfully predicts the influence of the surface receptor density, probe ligand valency, receptor–ligand affinity, and competitor/cofactor concentrations and affinities on superselective recognition. The scaling variable is a simple yet versatile tool to quantitatively tune the on/off threshold of superselective probes. We exemplify its application by reviewing and reinterpreting literature data for selected biological and biomedical interaction systems where superselectivity clearly is important.

Our guidelines can be deployed to generate a new mechanistic understanding of multivalent recognition events inside and outside cells and the downstream physiological/pathological implications. Moreover, the design rules can be harnessed to develop novel superselective probes for analytical purposes in the life sciences and for diagnostic/therapeutic intervention in biomedicine.

## Key References

- DubachevaG. V.; CurkT.; MognettiB. M.; Auzély-VeltyR.; FrenkelD.; RichterR. P.Superselective targeting using multivalent polymers. J. Am. Chem. Soc.2014, 136, 1722–1725.2440059110.1021/ja411138sPMC3919174(1)*First quantitative correlation of experiment and theory that demonstrates superselective targeting.*
- DubachevaG. V.; CurkT.; Auzély-VeltyR.; FrenkelD.; RichterR. P.Designing multivalent probes for tunable superselective targeting. Proc. Natl. Acad. Sci. U. S. A.2015, 112, 5579–5584.2590132110.1073/pnas.1500622112PMC4426472(2)*Demonstrates how superselective binding can be tuned through probe design to target a desired receptor density. Correlating experiments, computer simulations, and analytical theory, a simple scaling variable is established as a predictive tool to guide rational probe design.*
- DubachevaG. V.; CurkT.; FrenkelD.; RichterR. P.Multivalent recognition at fluid surfaces: the interplay of receptor clustering and superselectivity. J. Am. Chem. Soc.2019, 141, 2577–2588.3067601810.1021/jacs.8b12553(3)*Defines how receptor lateral mobility, relevant in cell membranes and facilitating receptor clustering, influences superselective recognition.*
- CurkT.; DubachevaG. V.; BrissonA. R.; RichterR. P.Controlling superselectivity of multivalent interactions with cofactors and competitors. J. Am. Chem. Soc.2022, 144, 17346–17350.3610360010.1021/jacs.2c06942PMC9523699(4)*Determines how cofactors and monovalent competitors modulate superselective recognition.*

## 1 Introduction

### 1.1 Definition of Superselectivity

**Figure 1:**
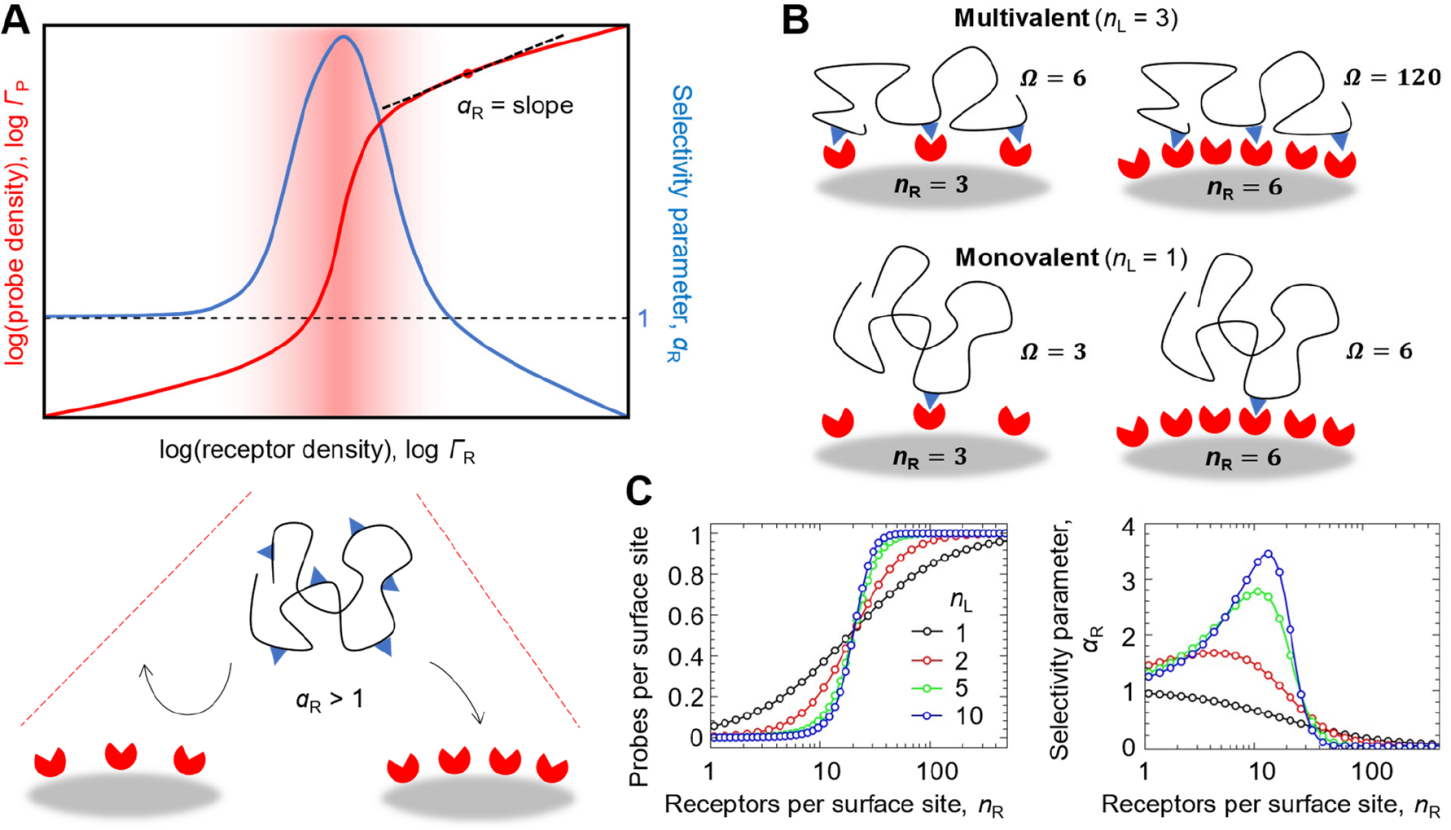
**Basic concepts of superselective binding (flexible probe).** (A) Representative dependencies of the probe surface density (Γ_P_) and selectivity parameter (α_R_) on the receptor surface density (Γ_R_; top) along with a schematic illustration of superselective recognition (bottom). (B) How combinatorial entropy leads to superselective binding (the number of binding states Ω changes sharply for multivalent but not for monovalent probes; *n*_R_ is the number of receptors within reach of the probe (*n*_R_ ∝ Γ_R_)). (C) Simple example (based on eqs 5 and 6) of how increasing probe valency (*n*_L_) amplifies the discrimination of surfaces by their comparative receptor densities (adapted with permission from ref (17); copyright (2018) John Wiley & Sons).

“Superselectivity” was coined by Martinez-Veracoechea and Frenkel^5^ for the ability of multivalent probes to sharply discriminate surfaces by their comparative densities of a given receptor (Figure 1A). This is in contrast to conventional “type” selectivity, which discriminates surfaces by their distinct receptor types. We use the terms “ligand” and “receptor” to denote binding partners on the probe and the surface, respectively, irrespective of whether they are ligands or receptors in a biological sense. For quantitative analyses, we define the selectivity parameter α_R_ as the slope in a double-logarithmic plot of probe density (Γ_P_) vs receptor density (Γ_R_)
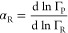
1where d ln Γ_R_ = dΓ_R_/Γ_R_ represents the relative change in receptor surface density and d ln Γ_P_ = dΓ_P_/Γ_P_ the associated relative change in the surface density of bound probes (Figure 1A). An interaction is superselective whenever α_R_ > 1, indicating that probe binding increases superlinearly with receptor density.

In later sections, we define superselectivity more broadly as a sharp (i.e., superlinear) change in binding as a function of any given parameter of interest. This broader definition, for example, encompasses well-known phenomena such as cooperative binding, where α_*c*_P__ = d ln Γ_P_/d ln *c*_P_ > 1 (with probe concentration *c*_P_) is equivalent to the well-known Hill coefficient being superior to 1.^6^

### 1.2 Superselectivity Is Not New, but the Underpinning Physics Has Long Remained Elusive

Superselective binding is neither new nor has it been invented by humans. The scientific literature is rich in reports of a superlinear increase of probe binding as a function of surface receptor density. In all reported cases, the probes bind their receptors multivalently, but the type of probe varies widely, including proteins,^7^ antibodies,^8,9^ biopolymers,^10,11^ viruses,^12−14^ liposomes, and nanoparticles.^15,16^

Superselective recognition plays important roles in basic cellular processes, including cell–cell and cell–extracellular matrix communication,^2,11^ immune recognition,^9^ cell membrane repair,^18^ and intracellular transport.^19,20^ It also contributes to pathological processes, e.g., the recognition of host cells by viruses.^13,14^ Arguably, superselective recognition is essential for the correct intracellular sorting of molecules and the spatiotemporal control of intracellular reactions, although much remains to be explored in this area. Superselective recognition also opens new avenues in biotechnology and medicine: it has the potential to add a new dimension to the selective targeting of cells (e.g., cancer cells, stem cells) for imaging, sorting, isolation, and treatment purposes.

Despite the pervasiveness of superselective recognition in biological systems, and its technological potential, the underpinning physical mechanisms have long remained elusive. In particular, the key role of combinatorial entropy as a “universal” driving force for superselective binding (Figure 1B,C) has been largely underappreciated and a quantitative theoretical treatment of the matter only emerged in the past decade.^2,5,17,21^

### 1.3 Basic Ingredients of Superselective Recognition

The minimal set of conditions required for binding to be superselective is the following:

i. **Multivalency:** The probe displays several (*n*_L_) ligands that recognize the receptors on the surface with a certain affinity and “type” selectivity. Although *n*_L_ > 1 is sufficient, superselectivity benefits from large numbers of binding sites (*n*_L_ ≫ 1).
ii. **Combinatorial entropy:** Multiple ligand–receptor pairs can form in many different combinations. This can be achieved most simply through conformational flexibility of the probe and/or the target surface. However, even for probes and surfaces with fixed ligand and receptor positions, respectively, the ligands and receptors can combine in many different combinations as long as their positions are disordered. Moreover, combinatorial entropy can be introduced in the case of regular ligand and receptor patterns via the promotion or interference of binding by free cofactors or competitors.
iii. **Low affinity:** The strength of individual ligand–receptor interactions (affinity *K*_d_) is weak, such that the probe (at probe concentration *c*_P_) does not attach strongly to a single receptor (*K*_d_ ≫ *c*_P_). Once the first bond is made, ligand–receptor proximity drives spontaneous formation of additional bonds (*K*_d_ < *n*_L_*c*_eff_, with *c*_eff_ being the effective concentration of receptors within reach of a ligand). In practice, suitable *K*_d_ values are in the micro/millimolar range.

We emphasize that these criteria do not place any stringent requirements on the chemical nature of the probe and the target surface and their ligands and receptors, respectively. Superselective targeting can be accomplished with many types of multivalent probes, as long as they are conformationally flexible or target a disordered surface (such as a fluid cell membrane with embedded receptors or an immobile surface with randomly positioned receptors). Obvious examples are probes based on flexible polymer scaffolds (linear or branched),^2,3^ nanoparticles,^5,15,16,22,23^ and liposomes and polymersomes.^24^ Likewise, “ligands” and “receptors” can be diverse, from any of the four classes of biomacromolecules (proteins, glycans, lipids, nucleic acids) or from synthetic analogues (e.g., host–guest chemistry). The design space, therefore, is vast providing plenty of opportunities for the development of superselective probes.

The criteria for superselective targeting, however, are distinct from conventional selective targeting. Natural antibodies and their analogues (aptamers, affimers, etc.) are selected for maximal affinity to their target receptor, with typical affinities in the nM or pM range. This violates criterion (iii). Strategies to develop recognition elements for superselective probes thus cannot follow the current selection paradigm and require new approaches.

### 1.4 Basic Mechanism Underpinning Superselective Binding

The essential feature of multivalent interactions is that the number of possible binding states depends sensitively on the number of available ligands and receptors. The number of distinct combinations Ω(*i*) to connect *n*_L_ ligands and *n*_R_ receptors via *i* bonds increases very sharply with *n*_L_ and *n*_R_ (Figure 1B). This gives rise to *combinatorial entropy* as an important contributor to multivalent interactions that must be explicitly considered. In the simplest approximation, the binding avidity scales with the number of possible binding states (*K*_av_ ∝ Ω). Ultimately, this entails a sharp rise in binding of multivalent probes as a function of receptor surface density (Figure 1C), i.e., superselective binding.

Below we review the basic theoretical foundation for superselective binding for the readers who are interested in a quantitative description of superselectivity. We assume that the multivalent probes are flexible such that any ligand in the probe can bind to any receptor within the area covered by the probe (Figure 1B). However, similar final results are obtained for probes with fixed ligand positions on surfaces with receptors that are randomly positioned^25^ (see the Supporting Information) or mobile.^3,26^ The number of ways Ω(*i*) to connect *n*_L_ ligands and *n*_R_ receptors via *i* bonds is the product of the number of possible ways to choose *i* ligands out of *n*_L_, the number of possible ways to choose *i* receptors out of *n*_R_, and the number *i*! (i.e., the factorial of *i*: *i*! = *i* × (*i* – 1) × ··· × 1) of possible ways to connect the ligands and receptors:
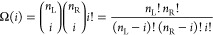
2The free energy *F* of the multivalent interaction is obtained by summing over all possible binding states using^2−5,17,27^
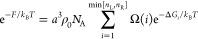
3where Δ*G*_*i*_ is the free energy of a *specific* configuration with *i* bonds and the prefactor *a*^3^ρ_0_*N*_A_ accounts for the size of the multivalent probe *a* and normalizes *F* with respect to the standard concentration ρ_0_ = 1 M (*N*_A_ is Avogadro’s number). Here, we do not consider cooperative allosteric effects^28^ and assume Δ*G*_*i*_ is proportional to the number of formed bonds
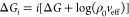
4where Δ*G* is the Gibbs free energy of the individual ligand–receptor interaction, which relates to the ligand–receptor dissociation constant, *K*_d_ = ρ_0_e^Δ*G*/*k*_B_*T*^. *v*_eff_ is the effective configurational volume that each unbound ligand can explore within the multivalent entity and determines the effective receptor concentration *c*_eff_ = *n*_R_/*v*_eff_. Here, we assume for simplicity that *v*_eff_ is a constant for any number of formed bonds and given by the molar volume of the probe, *v*_eff_ ≈ *a*^3^*N*_A_. In general, *v*_eff_ may be further tuned (e.g., by changing the length of the linker) and can be different for forming the first, second, and any higher number of bonds.^17,28^ While such variations influence the binding curve’s shape, they do not affect any scaling and tunability predictions discussed below.

The free energy *F* is related to avidity, which measures the overall strength of the multivalent interaction, expressed through the association constant *K*_av_ = ρ_0_^–1^e^–*F*/*k*_B_*T*^. The above equations provide a fundamental way to calculate the avidity of multivalent interactions but are also somewhat unwieldy. The theory can be greatly simplified if the receptors are mobile,^3^ the number of receptors is large, or individual bonds are weak, such that binding to different ligands can be considered to be uncorrelated (*n*_R_!/(*n*_R_ – *i*)! ≈ *n*_R_^*i*^); i.e., there is no local depletion of receptors.^17^Equations 2 and 3 can then be simplified, with avidity determined by^2,3,17^
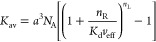
5For multivalent probes (*n*_L_ > 1), the avidity increases superlinearly with the receptor density Γ_R_ (where the number of receptors covered by the probe is *n*_R_ ≈ *a*^2^*N*_A_Γ_R_, i.e., a small change in Γ_R_ produces a large change in *K*_av_). This is the origin of superselective binding.

When multivalent probes bind to a surface or a membrane, the steric exclusion between probes typically leads to the well-known Langmuir adsorption isotherm with the surface occupancy given by
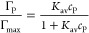
6Γ_max_ is the maximum surface probe density and *c*_P_ the probe concentration. The binding selectivity α_R_ (eq 1) is then characterized by the slope of the binding curve on a double logarithmic plot (Figure 1A).

Figure 1C illustrates how the binding curve becomes steeper and α_R_ increases by increasing the probe valency *n*_L_ for this simple model. Whereas at the outset we focused on selective targeting based on the receptor surface density, eq 5 implicates that equivalent superselective targeting can be achieved with respect to other parameters, such as the dissociation constant *K*_d_ (), the probe valency *n*_L_ (), or the effective configurational volume *v*_eff_. The effect of varying these parameters on superselective binding is the focus of this account. In addition, we will discuss other effects not considered in eq 5, e.g., monovalent competitors, binding via cofactors, and probe concentration.

## 2 Determinants of Superselective Binding

### 2.1 Experimental Platform Enabling Analysis of Superselective Binding, and Quantitative Correlation with Theory

#### 2.1.1 Experimental Platform

Determining the factors regulating superselective binding is challenging in real biological systems; teasing out the effect of individual parameters requires their controlled variation over a wide range, which is difficult in the complex environment of cells and tissues. Instead, we developed an experimental model interaction system in which salient parameters were quantitatively tunable while avoiding nonspecific probe/surface interactions.

**Figure 2:**
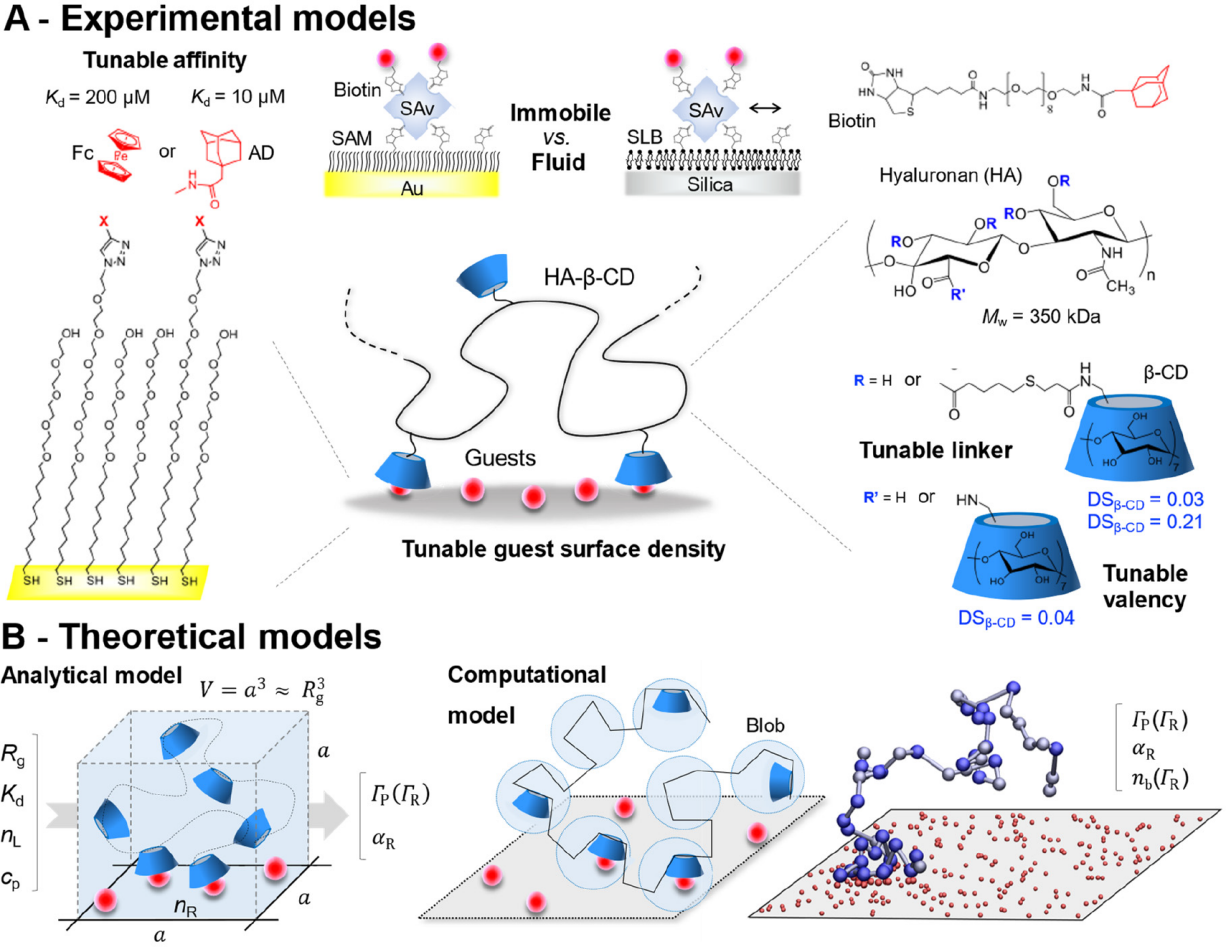
**Experimental and theoretical models to explore the determinants of superselective binding.** (A) Experimental models based on host–guest chemistry: probes were made with hyaluronan polymers and grafted β-CD “hosts” as ligands; target surfaces displayed “guests” (ferrocene, Fc, or adamantane, AD) as receptors at tunable densities, immobile (on self-assembled monolayers, SAMs; coupled covalently or via streptavidin (SAv)/biotin interactions) or in-plane mobile (on fluid supported lipid bilayers, SLBs; coupled via SAv/biotin). (B) Theoretical models: the analytical model (left) captures the spatial confinement of the polymer-bound ligands; computer simulations (right) additionally capture the valency and flexibility of the polymer-based probes (blue and gray spheres in the simulation snapshot represent polymer blobs with and without ligands, respectively).

The model was inspired by the naturally occurring multivalent interactions between the long, linear, and flexible polysaccharide hyaluronan (HA) and its cell surface receptors (Section 3.1). We used HA as a probe scaffold but replaced the native HA/receptor interactions by host/guest chemistry (Figure 2A).^1,2^ The β-cyclodextrin (β-CD)/guest system^29,30^ was well suited for the intended purpose owing to good β-CD solubility under physiological conditions, a wide affinity range (*K*_d_ = 0.01–10 mM, depending on the guest), and well-established conjugation chemistries facilitating tuning of valency *n*_L_ (via the degree of substitution, DS) and receptor density Γ_R_. With this model system, we could additionally probe the effects of in-plane receptor mobility,^3^ of the length of the linker connecting the hosts to the probe scaffold,^2^ and of the probe concentration.^2^ All probe and surface designs are illustrated in Figure 2A. We refer the reader to the original papers for details on their production and characterization^1−3^ and present the major findings of the interaction analyses in Section 2.2.

#### 2.1.2 Theoretical Models

To rationalize the obtained experimental results, we developed an analytical model (Figure 2B, left).^1−3^ The model is based upon the statistical mechanics approach outlined in Section 1.4 and ref (5) but additionally captures the ability of polymers to interpenetrate (which enhances the range of superselective binding^1^) and can also explicitly consider in-plane receptor mobility.^3^ Using just a few adjustable parameters, the model reproduced essential features of the experimental system.^1−3^

The analytical nature of this model is a major benefit: relevant parameters and their interdependencies can be identified easily, and predictions over a large multiparameter space can be made without expensive computational resources. A key outcome of the analytical model was the identification of the scaling variable *x*_S_ (Section 2.2) as a simple tool to tune the design of multivalent probes to target a desired superselectivity range.^2^

Certain aspects of the real interactions, however, were difficult to capture with a deliberately simple analytical model. In particular, the model assumed the effective configurational volume *v*_eff_ to be identical for each of the receptor/ligand bonds formed between the probe and the surface. In reality, for flexible polymers, *v*_eff_ gradually decreases as the number of bonds increases. Grand-canonical Monte Carlo computer simulations (Figure 2B, right) that explicitly considered the polymeric nature of the probe scaffold showed that the quality of superselective binding is even slightly higher than predicted by the analytical theory.^2^ The computational model also enabled visualization of receptor clustering upon probe binding on fluid surfaces,^3^ which is challenging in experiments owing to the limited spatial resolution of optical microscopy.

### 2.2 Factors Influencing Superselective Binding

This chapter describes the main insights obtained thanks to the developed experimental and theoretical tools. We start with the effect of probe valency, affinity, size, concentration, and receptor mobility on superselective recognition (Sections 2.2.1–2.2.3) and then extend to other factors such as the probe type, competitors, and cofactors (Sections 2.2.4 and 2.2.5).

#### 2.2.1 Affinity, Surface Receptor Density, Probe Ligand Valency, and Linker Variations

**Figure 3:**
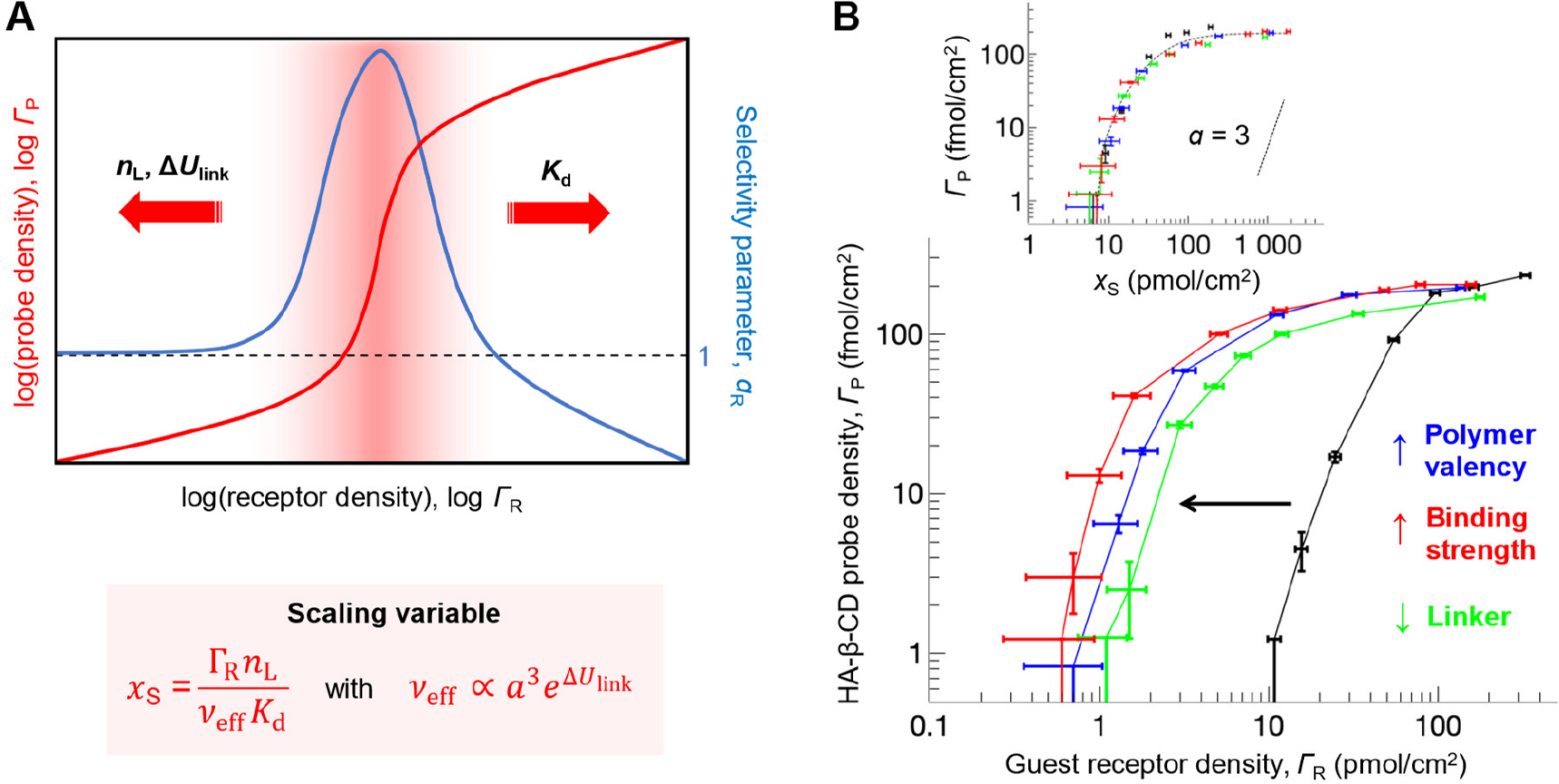
**Determinants of superselective binding (I): affinity (*K*_d_), probe ligand valency (*n*_L_), and variations in linkages (Δ*U*_l__i__n__k_).** (A) Schematic summary. (B) Illustrative data obtained with the host–guest system described in Figure 2A (adapted from ref (2)). Individual parameters were varied compared to the reference (black): affinity was increased 10-fold (red); ligand valency was increased 7-fold (blue); linkages of ligands to the probe scaffold were shortened (Δ*U*_link_ = −1.9 *k*_B_*T*; green). The inset demonstrates that all data collapse onto a master curve as a function of the scaling variable *x*_S_ (see ref (2) for details).

Using our experimental β-CD/guest model interaction system (Figure 2A), we characterized the selectivity of multivalent probes as a function of surface receptor (guest) density Γ_R_, for pairs of distinct β-CD/guest affinities (*K*_d_^Fc^ = 200 μM vs *K*_d_^AD^ = 10 μM), probe valencies (*n*_L_ ≈ 27 vs 187, corresponding to DS = 0.03 and 0.21 per HA disaccharide, respectively), and linker types (pentenoate vs amide bond) with all other parameters (including probe size, radius of gyration, *R*_g_ = 45 nm, and concentration, *c*_P_ = 120 nM) unchanged.^2^ While pronounced superselective binding was always observed (with α_R_ reaching maximal values above 3; Figure 3B, inset), the effects of the three varied parameters on the binding curve proved remarkably simple and similar: the shape was virtually unaffected, but the position shifted (to different extents) along the surface receptor density axis (Figure 3). The analytical model and computer simulations reproduced the experimental trends^2^ and explained the magnitude of the shifts quantitatively. A major result of this analysis is the scaling variable
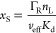
7

The scaling variable *x*_S_ is expected to faithfully predict the influence of receptor/ligand affinity (*K*_d_) and probe valency (*n*_L_) on the superselectivity range as long as the fractions of occupied receptors and ligands are low.^2^ More generally, any parameter affecting recognition purely ligand by ligand (i.e., without any cooperativity between ligands) shifts the superselectivity range but does not have a major impact on the quality of superselectivity. The effective configurational volume *v*_eff_ is here determined by the size of the probe (*a*^3^ ≈ *R*_g_^3^) and other effects that cannot be measured directly, such as the entropic cost of confining a polymer to a surface and ligands to the polymer.^2^ We here also include linkages between ligands and the probe scaffold (encompassed by the energy term Δ*U*_link_ = −1.9 *k*_B_*T*, when shifting from pentenoate to a simple amide; *v*_eff_ ∝ *a*^3^e^Δ*U*_link_/*k*_B_*T*^); although not experimentally shown, linkages between receptors and the surface should have an equivalent effect. *x*_S_ thus provides a simple yet effective theoretical tool for tuning the range of superselective surface receptor targeting.^2^

In addition, the scaling variable confers a more general meaning to superselective targeting: it implies that, if binding is superselective to one of the factors contained in *x*_S_, then it is superselective with the same quality to any of the other factors in *x*_S_. For example, surfaces with a suitably fixed receptor density can be used to superselectively discriminate nanoprobes by their valency,^31^ and probe–surface interactions with suitably fixed ligand and receptor presentations can be used to sharply discriminate interaction affinities (see Section 2.2.5).

#### 2.2.2 Probe Size and Concentration

**Figure 4:**
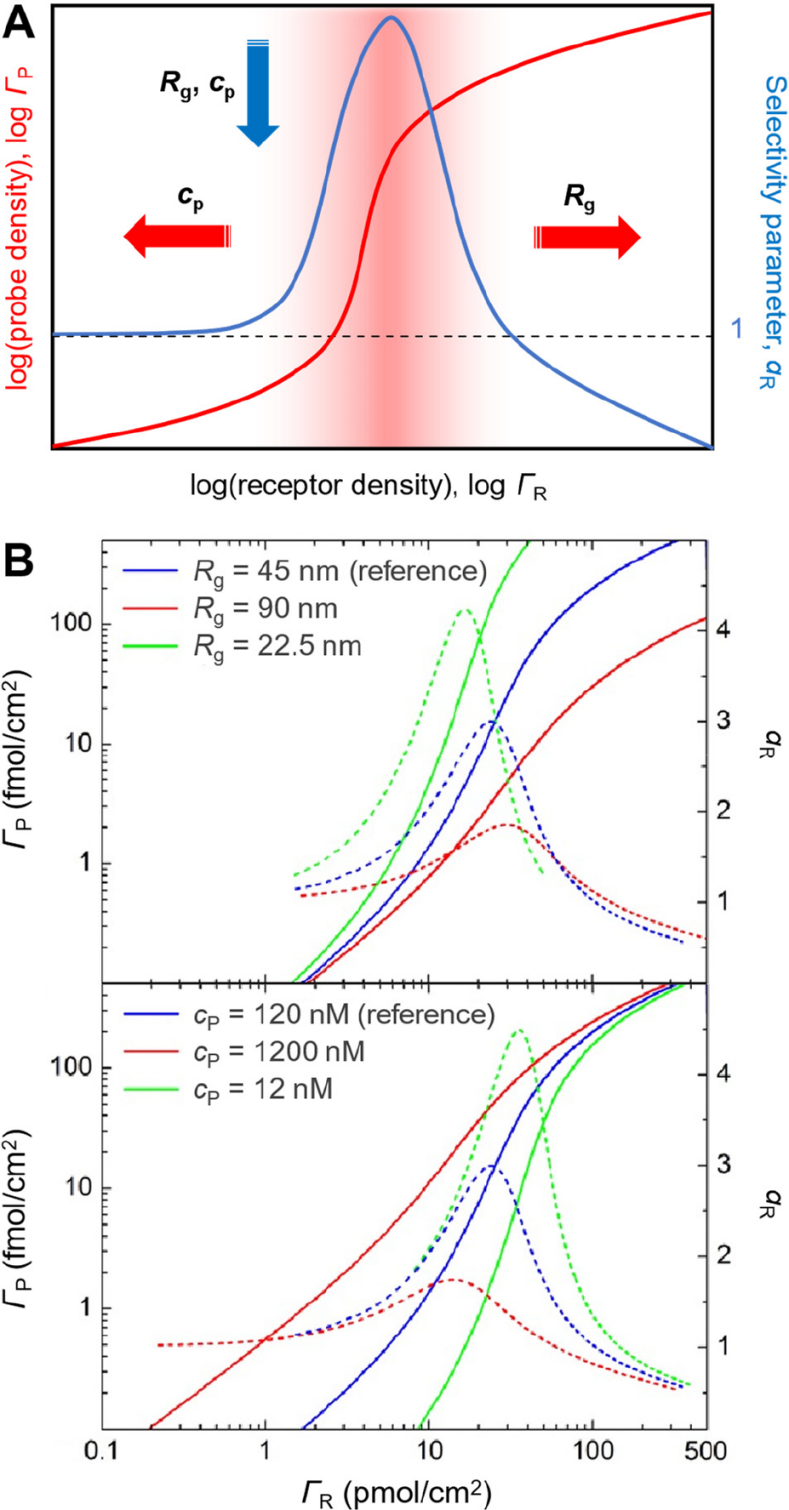
**Determinants of superselective binding (II): probe size (radius of gyration, *R*_**g**_) and concentration (*c*_P_).** (A) Schematic summary. (B) Predictions of the analytical model for Γ_P_ (solid lines) and α_R_ (dotted lines) vs Γ_R_ at a 2-fold reduced/increased size (top) and at a 10-fold reduced/increased concentration (bottom), with all other parameters kept identical (adapted from ref (2)).

The probe size *a* ≈ *R*_g_ and concentration *c*_P_ affect the binding profile in a more complex manner that is not satisfactorily encompassed by the scaling variable. The full analytical model predicts that not only the range but also the quality of the superselective binding is affected (Figure 4A).^2^ In essence, keeping the volume fraction of the probe low (ϕ ≈ *R*_g_^3^*N*_A_*c*_P_ ≪ 1) will maximize the effect of combinatorial entropy and thus the quality of superselectivity (as can be appreciated from eqs 5 and 6(17)). Moreover, an increase in probe size or concentration (at constant valency *n*_L_) shifts the superselectivity regime toward higher or lower receptor densities, respectively (Figure 4B). Predicted dependencies on concentration were qualitatively confirmed,^2^ yet the effect of size remains to be tested in experiments.

#### 2.2.3 Receptor Mobility

Experiments and computer simulations have demonstrated that multivalent probes essentially retain their superselective binding behavior at fluid surfaces.^3^ For interaction systems where the number of bonds formed always remains much smaller than the number of available ligands and receptors, the scaling variable *x*_S_ effectively describes how the superselective binding range can be tuned, irrespective of surface fluidity.

**Figure 5:**
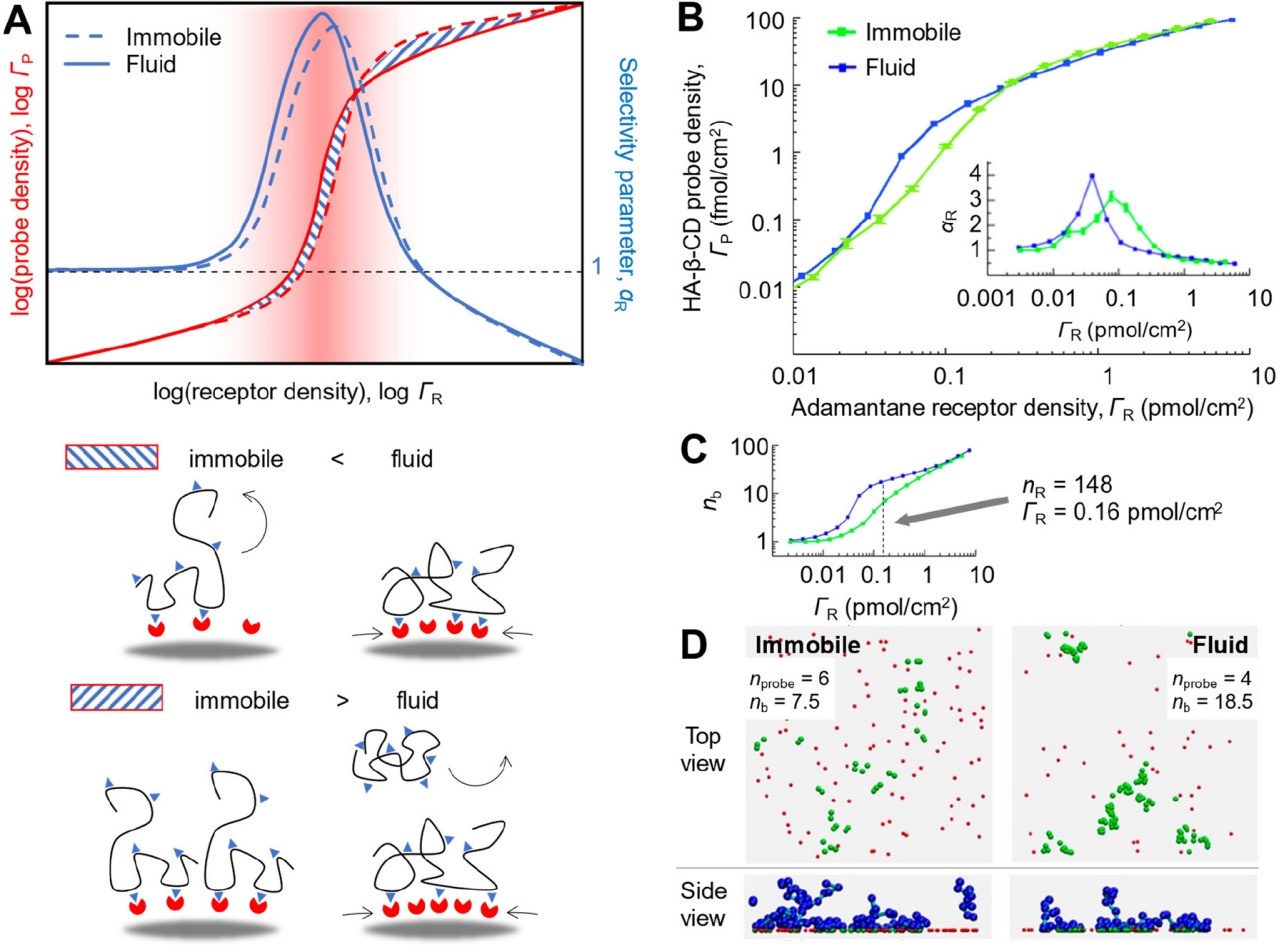
**Determinants of superselective binding (III): receptor lateral mobility.** (A) Schematic summary. (B–D) Illustrative data obtained with the soft-blob model for a polymer system shown in Figure 2B at *n*_L_ = 27, with dependencies of average binding valency (*n*_b_) on guest surface density (C) and snapshots illustrating receptor clustering on fluid surfaces (D; blue blobs and cyan joints represent polymers and red and green spheres correspond to unbound and bound receptors, respectively; in the top view, only the receptors are shown). Adapted from ref (3).

However, subtle changes to the binding curve occur for systems where binders can become saturated (Figure 5A), as best revealed by computer simulations (Figure 5B–D):^3^

i. Receptor mobility shifts the onset of superselective binding to lower average receptor densities and also enhances the quality of superselectivity (α_R,max_ increases). These effects are due to local accumulation of receptors (i.e., clustering) and the associated enhancement in combinatorial entropy and number of bonds formed.
ii. Probe binding is somewhat reduced at higher receptor densities because each probe binds more receptors on fluid surfaces on average, thus globally depleting receptors.

In this case, the full analytical model for in-plane mobile receptors (described in ref (3)) can be used to predict the influence of the probe characteristics on its superselective binding behavior.

#### 2.2.4 Probe “Scaffold” Type

The above trends were established with a multivalent linear polymer. However, similar effects are expected for multivalent probes based on other scaffold types, such as branched (including dendrimeric) polymers, solid particles, and liposomes. “Soft” probes are particularly versatile for superselective targeting because their intrinsic conformational flexibility inherently facilitates interactions with the target surface through many possible combinations of ligand–receptor interactions. Flexibility can be built into the probe in many possible ways, e.g., via long, flexible linkages on nanoparticles or surface fluidity in liposomes. However, even completely “rigid” probes (e.g., nanoparticles with ligands closely linked to their surface, or enveloped viruses with a rigid shell) can serve as superselective probes as long as the target surface is “soft” (e.g., fluid or with long, flexible linkages to receptors) or the surface receptors are randomly distributed.

Polymers (the linear polymers treated in the previous sections in particular) have an added benefit because they can interpenetrate, which slightly extends the region of superselective binding.^1−3^ However, we expect that quantitative predictions can be made for other probe scaffolds and target surfaces by adapting the theories presented here and elsewhere.^15,16,24^ This area of research clearly merits further exploration.

#### 2.2.5 Competitors and Cofactors

**Figure 6:**
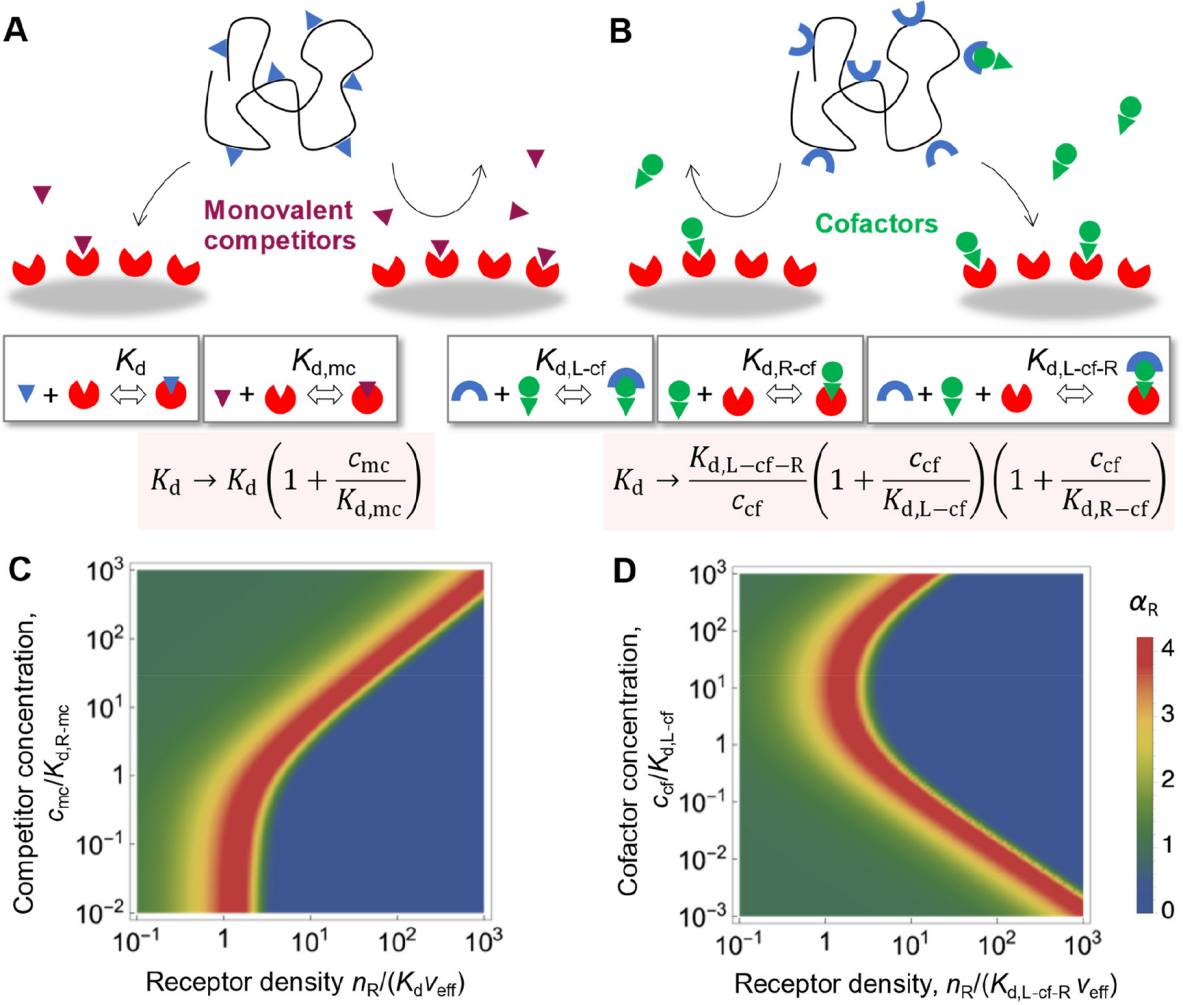
**Determinants of superselective binding (IV): monovalent competitors (concentration *c*_m__c_) and cofactors (concentration *c*_c__f_)**. (A, B) Schematic summaries. (C, D) Illustrative examples of the dependence of the selectivity parameter α_R_ on the concentration of receptors and monovalent competitors or cofactors, respectively (*c*_P_ = 10^–3^/(*a*^3^*N*_A_), *n*_L_ = 8, *K*_d,R-cf_ = 100*K*_d,L-cf_).

Rather than modifying the multivalent probe itself, adding monovalent binders as competitors is another, simple and thus attractive, avenue to modulate superselective binding (Figure 6A). An extended analytical model^4^ revealed that monovalent competitors (at concentration *c*_mc_) that bind to either receptors or ligands (with affinity *K*_d,mc_) effectively reduce the affinity between the receptors and the ligands on the multivalent probe, with the effective *K*_d_ becoming *K*_d_(1 + *c*_mc_/*K*_d,mc_). This effect can be exploited for “superselective” discrimination of competitor concentrations (Figure 6C). The generalized scaling variable *x*_S_, with *K*_d_ in eq 7 replaced by *K*_d_(1 + *c*_mc_/*K*_d,mc_), remains a simple tool to tune superselective binding.

In some cases, the binding between multivalent entities requires a soluble cofactor (Figure 6B). Similarly to monovalent competitors, the effect of cofactors (at solution concentration *c*_cf_) can be fully captured by using a generalized “affinity”.^4^ With cofactor dissociation constants *K*_d,L-cf_ and *K*_d,R-cf_ for ligands and receptors, respectively, the effective affinity becomes *K*_d_ = *K*_d,L-cf-R_/*c*_cf_(1 + *c*_cf_/*K*_d,L-cf_)(1 + *c*_cf_/*K*_d,R-cf_), where *K*_d,L-cf-R_ is the tripartite ligand/cofactor/receptor affinity constant. At small cofactor concentrations typical for biological systems, *c*_cf_ < *K*_d,L-cf_ and *c*_cf_ < *K*_d,R-cf_, we can approximate *K*_d_ = *K*_d,L-cf-R_/*c*_cf_ and thus changing the cofactor concentration has the same effect as changing the number of receptors *n*_R_ (cf. eq 5): the binding only depends on the generalized scaling variable *x*_S_ = Γ_R_*n*_L_*c*_cf_/(*K*_d,L-cf-R_*v*_eff_). Multivalent binding with cofactors can be exploited for simultaneous superselective discrimination of receptor surface densities and cofactors (Figure 6D).

## 3 Applying Superselectivity Concepts to Biology and Medicine

Multivalent interactions are commonplace in biology,^28^ and many multivalent systems also feature combinatorial entropy and low affinity. Superselectivity, therefore, is very likely to be important in many biological systems. The application of superselectivity concepts to biological systems is still in its infancy. A few examples shall illustrate how the “design rules” (Section 2.2) can be harnessed to understand how nature deploys superselective interactions, and for biomedical applications.

### 3.1 Superselective Recognition in Biological Systems

**Figure 7:**
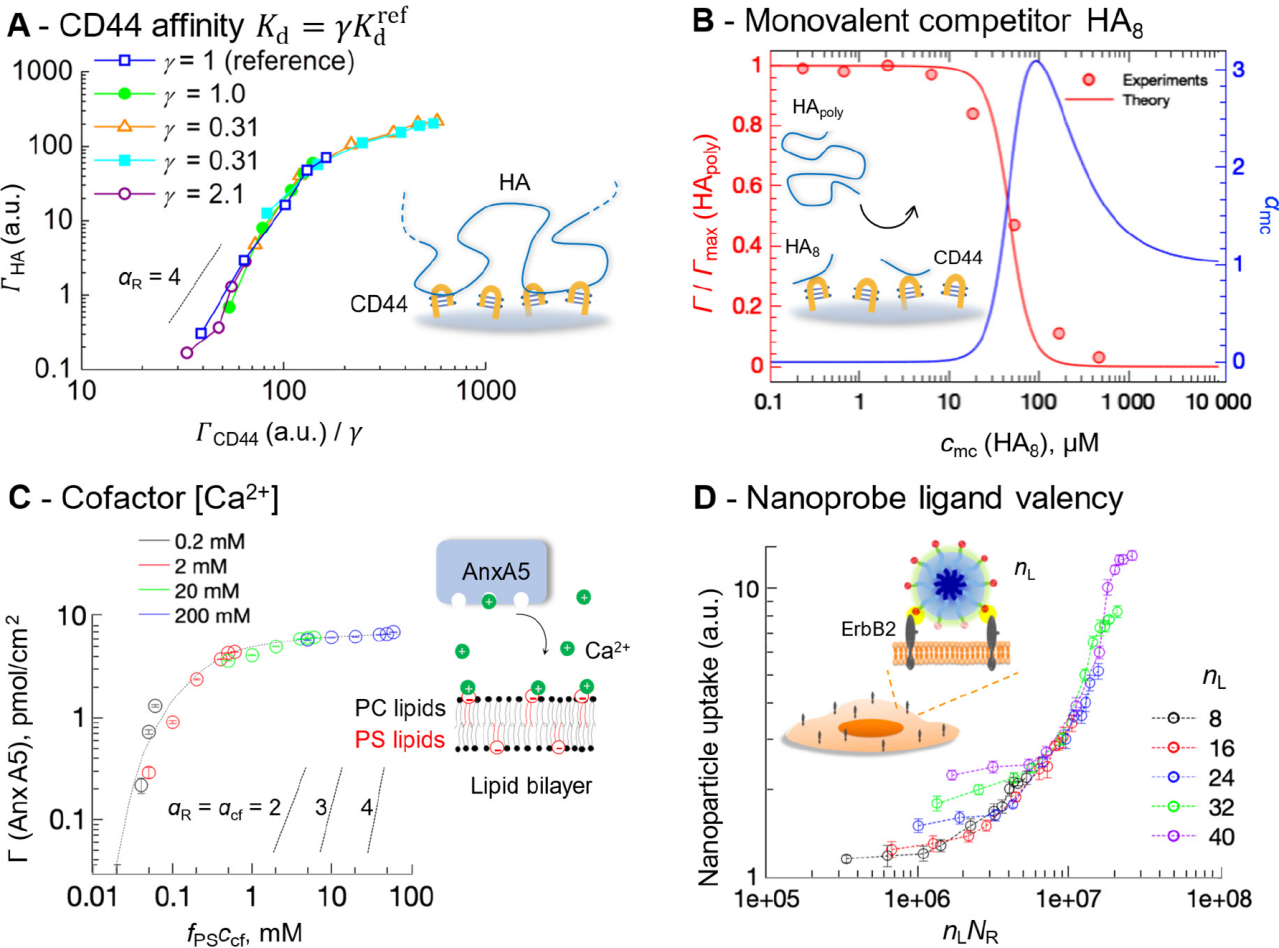
**Example applications of superselectivity concepts in biology and biomedicine.** (A) *Recognition of HA polysaccharides by its receptors*. Superposition of the binding curves for five distinct CD44 receptor variants shown in different colors (from ref (10)) onto a single master curve (by shifting with factor γ) demonstrates superselective binding (α_R,max_ ≈ 4) and enables quantitating relative differences in *K*_d_. Adapted from ref (2). (B) *Competition of HA polysaccharides (HA*_*poly*_*) by monovalent HA octasaccharides (HA*_8_*).* Red symbols - experimental data from ref (33); red line - prediction with the analytical model; the α_mc_ vs *c*_mc_ plot (blue line) demonstrates superselectivity with respect to the competitor concentration *c*_mc_; , with α_mc_ > 1. Adapted from ref (4). (C) *Recognition of anionic membranes by membrane repair protein annexin A5*. AnxA5 binding to supported lipid bilayers presenting phosphatidyl serine (PS; molar fraction *f*_PS_) in a background of phosphatidyl choline (PC) lipids, at different concentrations *c*_cf_ of the Ca^2+^ cofactor. The four sets of data at different Ca^2+^ concentrations collapse onto a master curve when plotted as a function of *f*_PS_*c*_cf_. Slopes with α values are included for reference. Adapted from ref (4). (D) *Cancer cell targeting using multivalent nanoparticles.* The particle uptake by the cell as a function of nanoparticle ligand valency (*n*_L_) (taken from Figure 4A in ref (34)) merges into a master curve when plotted as a function of *n*_L_*N*_R_, where *N*_R_ is the number of ErbB2 receptors per cell.

Cells exploit multivalent interactions between the polysaccharide HA and their surface receptors to probe their extracellular matrix environment. Superselective recognition of HA receptor surface density is evident for CD44^32^ and LYVE-1^11^ receptors. CD44 glycosylation modulates the receptor’s affinity for HA, to the point that two cells expressing CD44 at comparable levels but with distinct glycosylation exhibit pronounced vs virtually absent HA binding.^10^ Applying superselectivity concepts, we have shown that even a modest change in *K*_d_ is sufficient to “switch” HA binding on/off^2^ (Figure 7A).

Changes in HA presentation can also dramatically affect recognition. Simple affinity rescaling (Figure 6A) explains, for example, why oligosaccharides (e.g., formed as part of inflammatory responses) are potent inhibitors of HA polysaccharide binding to CD44 cell surface receptors^33^ despite their much lower comparative avidity.^4^ In fact, the interaction of HA polysaccharides with CD44 is superselective with respect to the concentration of oligosaccharide competitors (Figure 7B).

Another striking example is the superselective recognition of defective cell membranes by the protein annexin A5 (AnxA5). The protein binds anionic phospholipids and requires Ca^2+^ ions as a cofactor for membrane binding.^7^ Experiments with model membranes demonstrated that the membrane recognition by AnxA5 is superselective with respect to the concentrations of anionic lipids and Ca^2+^ cofactors, with α values up to approximately 4 (Figure 7C).^4^ This enables the protein to effectively respond to slight changes in the concentration of either of these two factors, which is crucial for its function as a membrane repair protein.^18^

### 3.2 Application of Superselectivity Concepts to Biomedicine

Understanding superselective behavior of multivalent systems also holds vast potential for medicinal chemistry as it suggests new approaches for the design of therapeutics intended for the efficient targeting for detection and treatment^9,34^ or inhibition^35,36^ of biological entities of interest (cells, viruses, bacteria).^37−39^ Carlson et al.^9^ provided an early yet striking example of superselective killing of tumor cells based on their overexpression of α_V_β_3_ integrins. Exploiting multivalent anti-α-galactosyl antibodies (including IgM with *n*_L_ = 10) to trigger complement-mediated cell death, along with a heterobifunctional cofactor to superselectively target the antibodies to the integrins, they achieved a selectivity superior to traditional monovalent high-affinity therapeutics.^9^ With an analogous approach, O’Reilly et al.^40^ demonstrated B cell targeting to be superselective with respect to the cofactor concentration.

Recently, Wang et al.^34^ demonstrated how nanoparticle ligand valency impacts multivalent interactions with breast cancer cells overexpressing the receptor ErbB2 at a range of densities (proportional to *n*_R_). In Figure 7D, we have replotted the data obtained with different particle valencies (*n*_L_ = 8–40) vs *n*_R_*n*_L_. That all data sets essentially collapse onto one master curve is indeed predicted by our scaling variable *x*_S_ (eq 7), and while not highlighted in the original study it demonstrates how superselective targeting can be precisely adjusted by tuning one or more parameters of the multivalent system. Recent examples also demonstrate how surfaces with tunable receptor densities can be exploited for the superselective detection of biopolymers, e.g., DNA by its level of methylation as a cancer biomarker.^41,42^

## 4 Conclusions and Perspectives

The above analyses of experimental data demonstrate the tangible benefits of superselectivity concepts. Simple “design rules” as defined in Section 2.2 should be considered in the conception of multivalent probes and in the analysis of past and future data to rationalize the implications of changes in the presentation of multivalent probes (e.g., concentration, size, valency), their receptors (e.g., affinity, surface density, and clustering), and the surrounding medium (e.g., competitors, cofactors) on recognition. Other factors have not been covered here yet provide additional dimensions to superselective recognition, such as the effect of mechanical force (leading to “hyperselectivity”^23^), macromolecular crowding,^43^ repulsive barriers (leading to “range selectivity”^44,45^), bulky competitors (to target surfaces with low receptor density^22^), surfaces with many distinct receptors,^26^ surfaces with a uniform receptor distribution,^46^ and the matching of ligand and receptor spatial patterns.^47^ Our design rules and the other factors generate a new mechanistic understanding of recognition events inside and outside cells and the downstream physiological/pathological implications. Such an understanding can be harnessed to develop novel superselective probes for analytical purposes in the life sciences and diagnostic/therapeutic intervention in biomedicine.

## Supporting Information Available

Additional information related to multivalent interactions with rigid probes (PDF)
