# Supplementary material for: Determinants of Superselectivity—Practical Concepts for Application in Biology and Medicine
Source: Acc Chem Res. 2023 Mar 14;56(7):729–39. doi: 10.1021/acs.accounts.2c00672 (PMC10077582; doi:10.1021/acs.accounts.2c00672)
Supplement: Supplementary file 1 — ar2c00672_si_001.pdf [file ar2c00672_si_001.pdf]

Supporting Information: Determinants of superselectivity – practical concepts for application in biology and medicine

Galina V. Dubacheva
Département de Chimie Moléculaire (DCM), UMR 5250, Université Grenoble Alpes, CNRS, 38000 Grenoble, France

Tine Curk
Department of Materials Science and Engineering, Johns Hopkins University, Baltimore, MD 21218

Ralf P. Richter
School of Biomedical Sciences, Faculty of Biological Sciences, School of Physics and Astronomy, Faculty of Engineering and Physical Sciences, Astbury Centre for Structural Molecular Biology, and Bragg Centre for Materials Research, University of Leeds, Leeds, LS2 9JT, United Kingdom

I. MULTIVALENT INTERACTIONS WITH RIGID PROBES

We consider a multivalent probe with rigidly attached inflexible ligands (e.g., a viral capsid) that is binding to an immobile surface with rigidly attached and randomly distributed receptors. Using statistical mechanics and ergodic hypothesis we will show that the expected value for the partition function (and consequently, the avidity constant between the probe and the surface) is independent of the mobility of the surface receptors for sufficiently large surfaces. Conversely, the variance in the partition function (i.e., the variance in the probe–surface binding free energy depending on the probe position) does depend on the surface fluidity, probe flexibility and ligand/receptor flexibility. For rigid probes on rigid surfaces, the increased variance in binding strength for immobile surfaces can lead to heterogenous adsorption which broadens the binding curve and thus reduces the selectivity parameter α compared to mobile surfaces or flexible probes.

To calculate the probe–surface interaction, we aim to evaluate the partition function, which is a sum over all possible probe positions on the surface. A rigid probe with attached inflexible ligands is positioned at $\mathbf{r}_\mathrm{p}$ close to the surface. The surface site has an area A and contains N randomly distributed receptors at average surface molar density $\Gamma_\mathrm{R} = \langle N\rangle/(AN_\mathrm{A})$, with N_A Avogadro's constant. Note that the size of A can be chosen arbitrarily. A is usually chosen as the surface area occupied by the probe, which facilitates applying the Langmuir adsorption model to this system.

The receptors are anchored at positions $\mathbf{r}_\mathrm{R}$ and are distributed according to a probability distribution $p(N, \mathbf{r}_\mathrm{R}^N)$, which takes into account possible receptor-receptor and other interactions and determines the probability that N receptors are located within a site at positions $\mathbf{r}_\mathrm{R}^N$. This distribution maps to a specific receptor–receptor radial distribution function. Note, receptors are not necessarily anchored on a 2D surface and could be positioned within some 3D space.

At this point, the orientation of the probe is kept fixed such that the positions of all ligands ($\mathbf{r}_\mathrm{L}$) relative to the probe center of mass position, $\mathbf{r}'_\mathrm{L} = \mathbf{r}_\mathrm{L} - \mathbf{r}_\mathrm{p}$, are constant. For a specific probe position $\mathbf{r}_\mathrm{p}$ and specific positions of receptors $\mathbf{r}_\mathrm{R}^N$, a bond can only form if a ligand–receptor pair is sufficiently close together. The bond formation probability is determined by the bond strength and by the ligand–receptor distance, which is captured by the free energy of the ligand–receptor bonding configuration $F_i(\mathbf{r}_\mathrm{R}^N, \mathbf{r}_\mathrm{p})$. For rigid systems, the distance within which bonding can occur is very small, but it is always non-zero due to thermal fluctuations. The partition function which takes into account all possible ways to form bonds between a probe at position $\mathbf{r}_\mathrm{p}$ and the surface is

$$Q_\mathrm{b}(\mathbf{r}_\mathrm{R}^N, \mathbf{r}_\mathrm{p}) = \sum_i e^{-\beta F_i(\mathbf{r}_\mathrm{R}^N, \mathbf{r}_\mathrm{p})} , \tag{S1}$$

where the sum proceeds over all possible ways to form bonds between the probe and the surface, $F_i(\mathbf{r}_\mathrm{R}^N, \mathbf{r}_\mathrm{p})$ is the free energy of a specific bonding configuration and $\beta = 1/(k_\mathrm{B}T)$. At this point, we make no assumptions on the details of the multivalent interactions. We only require that at least one bond is formed for the probe to be considered attached to the surface. In a rigid system, this partition function

will be non-negligible only when at least one ligand–receptor pair is sufficiently close together to form a bond. In other words, $F_i(\mathbf{r}_\mathrm{R}^N, \mathbf{r}_\mathrm{p})$ favors bond formation only for probes that are close to the surface and for specific probe positions. Note, however, that $F_i(\mathbf{r}_\mathrm{R}^N, \mathbf{r}_\mathrm{p})$ depends only on the relative positions between ligands and receptors.

To obtain the binding strength of the probe to the surface we must integrate Eq. (S1) over the volume V above the surface A to obtain the partition function,

$$Q_\mathrm{b}(\mathbf{r}_\mathrm{R}^N, A) = \rho_0 N_\mathrm{A} \int_V \mathrm{d}\mathbf{r}_\mathrm{p} Q_\mathrm{b}(\mathbf{r}_\mathrm{R}^N, \mathbf{r}_\mathrm{p}) \ , \tag{S2}$$

where the prefactor $\rho_0 N_\mathrm{A}$ determines the reference unit and we use standard concentration $\rho_0 = 1\,\mathrm{M}$ as the reference. This partition function determines the avidity constant of binding the probe to a specific surface site with area A,

$$K_\mathrm{av} = \frac{1}{8\pi^2 \rho_0} \int_\Omega Q_\mathrm{b}(\mathbf{r}_\mathrm{R}^N, A) \mathrm{d}\Omega \ , \tag{S3}$$

where the partition function must be integrated over all probe orientations Ω and normalized by the spherically symmetric case, $\int \mathrm{d}\Omega = 8\pi^2$, because the chemical potential reference ρ_0 assumes no angular component.

Obviously, the partition function [Eq. (S2)] depends on the exact number and positions of receptors. However, we are interested in the average $\langle K_\mathrm{av} \rangle$ over a large surface. In the limit of a very large surface, with a total number of sites M, we can consider an ensemble of surface sites that have different numbers of receptors N and different receptor positions $\mathbf{r}_\mathrm{p}^N$ determined by the probability distribution $p(N, \mathbf{r}_\mathrm{R}^N)$. The average partition function is

$$\langle Q_\mathrm{b}(A) \rangle = \frac{1}{M} \sum_{m=1}^{M} Q_\mathrm{b}(\mathbf{r}_\mathrm{R}^N, A) \ , \tag{S4}$$

where m denotes a specific surface site and both the number N and positions $\mathbf{r}_\mathrm{R}^N$ of receptors depend on the site m. For a large ensemble of sites, the sum over all surface sites with specific receptor positions is equal to the integral over all possible receptor positions weighted by the probability of a specific realization, and summed over all possible number of receptors,

$$\frac{1}{M} \sum_{m=1}^{M} [\cdot] = \sum_{N=1}^{\infty} \int \mathrm{d}\mathbf{r}_\mathrm{R}^N p(N, \mathbf{r}_\mathrm{r}^N) [\cdot] \ , \tag{S5}$$

where $[\cdot]$ denotes any quantity or function. Note that the probability distribution, $p(N, \mathbf{r}_\mathrm{R}^N)$ drops to zero above a certain N so that the number of elements in the sum that must be considered is finite. For example, if the receptors are non-interacting (ideal), the receptor positions are distributed uniformly at random and the distribution $p(N, \mathbf{r}_\mathrm{r}^N) = A^{-N} P(N)$ where $P(N)$ is a Poisson distribution in N.

The average partition function can thus be written as integral

$$\langle Q_\mathrm{b}(A) \rangle = \sum_{N=1}^{\infty} \int \mathrm{d}\mathbf{r}_\mathrm{R}^N p(N, \mathbf{r}_\mathrm{R}^N) Q_\mathrm{b}(\mathbf{r}_\mathrm{R}^N, A) \ , \tag{S6}$$

We insert Eq. (S2) into Eq. (S6) and swap the integration order to obtain

$$\langle Q_\mathrm{b}(A) \rangle = \rho_0 N_\mathrm{A} \int_V \mathrm{d}\mathbf{r}_\mathrm{p} \sum_{N=1}^{\infty} \int \mathrm{d}\mathbf{r}_\mathrm{R}^N p(N, \mathbf{r}_\mathrm{R}^N) Q_\mathrm{b}(\mathbf{r}_\mathrm{R}^N, \mathbf{r}_\mathrm{p}) \ . \tag{S7}$$

The inner sum in the above equation is equal to the partition function at the fixed probe position over all possible receptor positions, i.e., this is the partition function of a system with mobile receptors that obeys the receptor distribution $p(N, \mathbf{r}_\mathrm{r}^N)$,

$$Q_\mathrm{mobile}(\mathbf{r}_\mathrm{p}) = \sum_{N=1}^{\infty} \int \mathrm{d}\mathbf{r}_\mathrm{R}^N p(N, \mathbf{r}_\mathrm{R}^N) Q_\mathrm{b}(\mathbf{r}_\mathrm{R}^N, \mathbf{r}_\mathrm{p}) \ . \tag{S8}$$

Since $F_i(\mathbf{r}_\mathrm{R}^N, \mathbf{r}_\mathrm{p})$, and consequently $Q_\mathrm{b}(\mathbf{r}_\mathrm{R}^N, \mathbf{r}_\mathrm{p})$, only depend on the relative positions between ligands and receptors [see Eq. (S1)], the mobile partition function is the same for all probe positions $\mathbf{r}_\mathrm{p}$ at a specific distance z from the surface. $Q_\mathrm{mobile}(\mathbf{r}_\mathrm{p})$ does depend on z, but not on the lateral position of the probe relative to the surface, and thus also does not depend on the location of the surface site m. The mobile partition function per site A is, analogous to Eq. (S2),

$$Q_\mathrm{mobile}(A) = \rho_0 N_\mathrm{A} \int_V Q_\mathrm{mobile}(\mathbf{r}_\mathrm{p})\mathrm{d}\mathbf{r}_\mathrm{p} = \rho_0 N_\mathrm{A} A \int_{z=0}^{\infty} Q_\mathrm{mobile}(\mathbf{r}_\mathrm{p})\mathrm{d}z \;, \tag{S9}$$

Therefore, Eq. (S7) is equal to Eq. (S9) meaning that the average partition function, and thus the average avidity constant, of immobile, rigid surfaces is equal to that of mobile or fluid receptor surfaces.

$$\langle Q_\mathrm{b}(A)\rangle = Q_\mathrm{mobile}(A) \;. \tag{S10}$$

Note, to get the avidity from $Q_\mathrm{mobile}(A)$ and $\langle Q_\mathrm{b}(A)\rangle$, we also need to integrate over probe orientations, but these integrals are the same for mobile and immobile surfaces [Eq. (S3)].

This result shows that rigid and mobile receptors yield exactly the same expected value for the partition function of binding, and thus the same average avidity of binding. The only condition is that both the rigid surface and the mobile surface are (in the absence of probes) characterized by the same receptor distributions $p(N, \mathbf{r}_\mathrm{R}^N)$, i.e., in the absence of probes, the receptor radial distribution functions must be the same for mobile and rigid surfaces.

Conversely, the variance in avidity between different surface sites does depend on the surface mobility. Immobile surfaces can exhibit large spatial variance in avidity, while on mobile surfaces every surface site has the same avidity. High variance between surface sites leads to heterogenous adsorption which reduces the selectivity parameter α. However, even for rigid probes on immobile surfaces the variance can be small provided that the receptor density is sufficiently high such that most lattice sites allow at least one probe configuration where multiple bonds are formed. Thus, even when probes are rigid and the surface is immobile, the system can still be super-selective provided that the variance in K_av depending on the probe position is not too large. See Ref. [1] for a quantitative comparison between fluid and immobile surfaces in the case of colloids, and SI of Ref. [2] for the derivation of super-selective binding at fluid surfaces.

[1] Tine Curk, Urban Bren, and Jure Dobnikar. Bonding interactions between ligand-decorated colloidal particles. *Molecular Physics*, 116(21-22):3392–3400, 2018.

[2] Tine Curk, Jure Dobnikar, and Daan Frenkel. Optimal multivalent targeting of membranes with many distinct receptors. *Proc. Natl. Acad. Sci. U.S.A.*, 114:7210–7215, 2017.
